# Retraction of “Universal theranostic CRISPR/Cas13a RNA-editing system for glioma”

**DOI:** 10.7150/thno.96658

**Published:** 2024-04-06

**Authors:** Ye Wu, Yunfei Wang, Junhu Zhou, Jianhao Wang, Qi Zhan, Qixue Wang, Eryan Yang, Weili Jin, Fei Tong, Jixing Zhao, Biao Hong, Junrui Liu, Chunsheng Kang

**Affiliations:** 1 Tianjin Neurological Institute, Tianjin Medical University General Hospital, Key Laboratory of Post-neurotrauma Neuro-repair and Regeneration in Central Nervous System, Ministry of Education, Tianjin City, Tianjin 300052, China.; 2 Department of Dermatovenereology, Tianjin Medical University General Hospital, Tianjin 300052, China.; 3 Tianjin Key Laboratory of Composite and Functional Materials, School of Materials Science and Engineering, Tianjin University, Tianjin, China.; 4 College of Pharmacy, Kunming Medical University, Yunnan, China.

We, the authors, are writing to retract our publication "Universal theranostic CRISPR/Cas13a RNA-editing system for glioma" (DOI: 10.7150/thno.84429, Theranostics, 2023; 13(15): 5305-5321). After thorough analysis, we discovered that the immunohistochemistry staining images in Figure 6 l and 6L were reused from a prior publication. We have identified all the raw data, but changing the representative images will not change their corresponding statistical analysis. Despite the fact that this resulted from ignorance, we elected to retract this publication since we are aware that it will damage the integrity of the scientific research. We sincerely apologize for any inconvenience caused by the retraction process of our paper. Our team take full responsibility and deeply regret the error.

